# A single dose of a neuron-binding human monoclonal antibody improves brainstem NAA concentrations, a biomarker for density of spinal cord axons, in a model of progressive multiple sclerosis

**DOI:** 10.1186/s12974-015-0303-y

**Published:** 2015-04-29

**Authors:** Bharath Wootla, Aleksandar Denic, Jens O Watzlawik, Arthur E Warrington, Moses Rodriguez

**Affiliations:** Department of Neurology, Mayo Clinic, 200 1st Street SW, Rochester, MN 55905 USA; Department of Immunology, Mayo Clinic, 200 1st Street SW, Rochester, MN 55905 USA

**Keywords:** Multiple sclerosis, Theiler’s murine encephalomyelitis virus, MRS, *N*-acetyl-aspartate, Brainstem, Axons

## Abstract

**Background:**

Intracerebral infection of susceptible mouse strains with Theiler’s murine encephalomyelitis virus (TMEV) results in chronic demyelinating disease with progressive axonal loss and neurologic dysfunction similar to progressive forms of multiple sclerosis (MS). We previously showed that as the disease progresses, a marked decrease in brainstem *N*-acetyl aspartate (NAA; metabolite associated with neuronal integrity) concentrations, reflecting axon health, is measured. We also demonstrated stimulation of neurite outgrowth by a neuron-binding natural human antibody, IgM12. Treatment with either the serum-derived or recombinant human immunoglobulin M 12 (HIgM12) preserved functional motor activity in the TMEV model. In this study, we examined IgM-mediated changes in brainstem NAA concentrations and central nervous system (CNS) pathology.

**Findings:**

^1^H-magnetic resonance spectroscopy (MRS) showed that treatment with HIgM12 significantly increased brainstem NAA concentrations compared to controls in TMEV-infected mice. Pathologic analysis demonstrated a significant preservation of axons in the spinal cord of animals treated with HIgM12.

**Conclusions:**

This study links drug efficacy of slowing deficits with axon preservation and NAA concentrations in the brainstem in a model of progressive MS. HIgM12-mediated changes of NAA concentrations in the brainstem are a surrogate marker of axon injury/preservation throughout the spinal cord. This study provides proof-of-concept that a neuron-reactive human IgM can be therapeutic and provides a biomarker for clinical trials.

**Electronic supplementary material:**

The online version of this article (doi:10.1186/s12974-015-0303-y) contains supplementary material, which is available to authorized users.

## Findings

### Introduction

Multiple sclerosis (MS) is an inflammatory demyelinating disease of the central nervous system (CNS). The pathogenesis of the disease also involves axonal injury. Currently, there are no FDA-approved drugs that protect neurons and axons from degeneration. We previously showed that a human mAb of the IgM isotype, human immunoglobulin M 12 (HIgM12), bound to the surface of neurons, supported robust neurite extension when presented as substrate, and overrode the neurite extension inhibition of CNS myelin [1]. The Theiler’s murine encephalomyelitis virus (TMEV)-induced model of human demyelinating disease has been used extensively in our laboratory as a therapeutic drug discovery platform. This model was used to screen for the remyelination promoting human IgM, HIgM22, that recently completed phase I clinical trial in patients with MS [2]. Using this model, we also showed that a single dose of HIgM12 improved clinical disease course of virus-infected mice beginning at 2 weeks following treatment, which persisted for 8 weeks [3]. A recombinant form of human IgM12 showed identical biological properties to the serum-derived antibody, that is, induced neurite extension and neuronal protection *in vitro* [4] and improved neurologic function of TMEV-infected mice *in vivo* [5].

**Figure 1:**
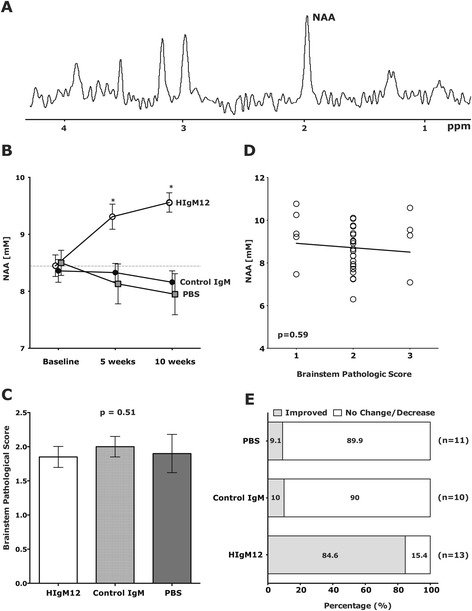
A single dose of neuron-binding human antibody improves NAA concentrations in the brainstem. **(A)** A representative 300 MHz, ^1^H spectra collected at the mouse brainstem. *N*-acetyl-aspartate (NAA) marked to the right is the dominant peak. **(B)** Groups of 9 to 13 SJL/J mice at 90 days post-TMEV infection, the time when NAA levels begin to fall and large-axon loss is detectable, were given a single 100 μg dose of HIgM12, isotype IgM control, or PBS i.p*.* MRS at the brainstem was collected before and at 5 and 10 weeks following the treatment. NAA concentrations were calculated from the spectra. Compared to pre-treatment, NAA increased in the HIgM12-treated group after 10 weeks (*P* < 0.001). NAA in the control IgM-treated group did not change (*P* = 0.188); in the PBS-treated group, it decreased after 10 weeks (*P* = 0.027). **(C)** After the last MRS measurements, mice were sacrificed, brains removed and processed for pathology analysis. Brainstem pathological scores (means ± SEM) were similar across treatment groups (*P* = 0.51). **(D)** Brainstem NAA concentrations plotted against the brainstem pathological score showed no correlation (*P* = 0.59). **(E)** Changes in the individual NAA concentrations were calculated at the final time point *versus* before treatment. NAA levels were considered improved if the difference (NAA^10wk^ − NAA^before^) was higher than or equal to 2 × baseline SEM. Fischer’s exact test was performed, and the control IgM-treated group did not differ from PBS-treated group (*P* = 0.388). The HIgM12-treated group differed from both control groups (*vs* control IgM, *P* = 0.0001 and *vs* PBS, *P* = 0.001).

Changes in brain metabolites measured by magnetic resonance spectroscopy (MRS) reflect neurological pathology. *N*-acetyl-aspartate (NAA), a derivative of aspartic acid, is the second most abundant free amino acid metabolite in nervous tissue [6] after glutamate and provides a prominent peak in MRS readouts *in vivo*. A representative prominent peak of NAA obtained from spectra collected at the mouse brainstem is shown (Figure 1A). Reduced NAA concentrations correlated with reduced axonal numbers in lesions of secondary progressive MS (SPMS) patients [7], primary progressive MS (PPMS), and relapsing remitting MS (RRMS) [8]. Most studies use brain MRS readouts for diagnostic purposes. Although rare, MRS of the spinal cord (SC) has been performed in humans [9] and rats [10]. Studies of MS patients’ showed reduced NAA levels at cervical SC compared to control humans [11]. We recently reported that NAA concentrations in the brainstem of TMEV-infected Swiss Jim Lambert (SJL) mice mimic disease progression [12].

Treatment of TMEV-infected mice with HIgM22 promoted remyelination that reached maximal levels by 5 weeks after treatment and remained at the same level 10 weeks post-treatment [13]. This suggests that if the degree of remyelination improved, then the axons themselves that are wrapped should be preserved as well. We hypothesized that MRS at the brainstem may be a viable endpoint in clinical trials designed to preserve or protect axons in the spinal cord. To test this idea, we treated TMEV-infected mice with the neuron-binding HIgM12 antibody and asked whether changes in NAA concentrations serve as a reliable endpoint marker.

## Methods

### Ethics statements

The Mayo Clinic Institutional Animal Care and Use Committee (IACUC) approved all animal protocols used in this study.

### Theiler’s virus model of demyelination

Demyelinating disease was induced in 8-week-old SJL/J mice by intracerebral injection of 10 μl containing 2.0 × 10^5^ plaque-forming units of Daniel’s strain TMEV. This resulted in >98% incidence of infection with rare fatalities [14].

### Antibodies and treatment

HIgM12 was used as the treatment. A serum-derived IgM non-reactive to neurons was used as control. SJL mice at 90 dpi were treated with a single 100 μg intraperitoneal dose of HIgM12 or controls (control IgM, phosphate-buffered saline (PBS)).

### Magnetic resonance spectroscopy

MRS was performed using a Bruker Avance 300 MHz (7 T) vertical bore NMR spectrometer (Bruker Biospin, Billerica, MA, USA). During data acquisition, animal core temperatures were maintained at 37°C by a flow of warm air. Inhalational isoflurane anesthesia 1.5% to 2.5% in oxygen was delivered via nose cone. MRS data were obtained, and NAA concentrations quantified from a (2.5 × 2.5 × 2.5) mm^3^ voxel (15.625 μl), placed over the brainstem as reported previously [12]. MRS data was collected from each mouse before treatment and at 5 and 10 weeks later. The same investigator selected all voxels based on anatomical landmarks to maintain strict uniformity. Bruker’s VSEL sequence, an implementation of the standard PRESS sequence, was used for voxel-based spectroscopy, with built-in water suppression pulses.

### Spinal cord morphometry

Spinal cord morphometry was performed according to the sampling scheme reported previously [12]. We found that a consistent 1-μm cross-section from any level of the spinal cord can be used for axon frequency analysis; however, the T6 level was selected because it is the smallest thoracic spinal cord cross section and has a high white matter to gray matter ratio [15]. A 1-μm araldite-embedded section was cut from a mid-thoracic (T6) block for axonal analysis. Cross sections were stained with 4% *p*-phenylenediamine to visualize the myelin sheaths. Approximately 400,000 μm^2^ of white matter was sampled from each mouse. Absolute myelinated axon numbers were calculated as reported in [16]. Data were represented as the absolute number of all axons sampled per mid-thoracic spinal cord section. All gradings were performed on coded sections without the knowledge of the experimental group.

### Brain pathology

Brain pathology was assessed after the last MRS measurement, using our previously described technique [17]. Following perfusion with Trump’s fixative, we made two coronal cuts in the intact brain at the time of removal from the skull (one section through the optic chiasm and a second section through the infundibulum). As a guide, we used the *Atlas of the Mouse Brain and Spinal Cord* corresponding to sections 220 and 350, page 6 [18]. This resulted in three blocks that were then embedded in paraffin and allowed for a systematic analysis of the pathology of the cortex, corpus callosum, hippocampus, brainstem, striatum, and cerebellum. Resulting sections were then stained with hematoxylin and eosin. Pathological scores were assigned without knowledge of experimental group to the following areas of the brain: cortex, corpus callosum, hippocampus, brainstem, striatum, and cerebellum. Each area of the brain was graded on a five-point scale as follows: 0, no pathology; 1, no tissue destruction but only minimal inflammation; 2, early tissue destruction (loss of architecture) and moderate inflammation; 3, definite tissue destruction (demyelination, parenchymal damage, cell death, neurophagia, neuronal vacuolation); and 4, necrosis (complete loss of all tissue elements with associated cellular debris). Meningeal inflammation was assessed and graded as follows: 0, no inflammation; 1, one cell layer of inflammation; 2, two cell layers of inflammation; 3, three cell layers of inflammation; and 4, four or more cell layers of inflammation. The area with maximal tissue damage was used for assessment of each brain region. The data were expressed as mean ± standard error of the mean.

### Data analysis and statistics

Data for NAA concentrations and axon-count analysis were compared by Student’s *T* test if normally distributed or by Mann-Whitney rank sum test if non-normally distributed. Groups greater than two were subjected to one-way ANOVA analysis when they were normally distributed or to Kruskal-Wallis ANOVA on ranks when non-normally distributed. In all analyses, *P* < 0.05 was considered as statistically significant. Correlation coefficients between paired sets of data were determined using the Pearson product moment correlation. Fisher’s exact test was used to compare animals with improved NAA *versus* those with no change/decrease in NAA concentrations.

## Results

To confirm whether HIgM12 preserves neuronal health in the spinal cords of TMEV-infected mice, we used brainstem NAA concentrations measured by MRS as a biomarker. We elected to treat TMEV-infected mice at 90 dpi. At this time, maximal demyelination coincides with a drop in NAA concentrations. Following collection of baseline NAA concentrations at 90 dpi, three groups of 10 to 13 mice received a single intraperitoneal dose of HIgM12 (100 μg), control human IgM (100 μg), or saline (PBS). MRS measurements were repeated at 5 and 10 weeks post-treatment. In the control IgM-treated group, we found no significant differences in NAA concentrations between baseline and later time points (*P* = 0.74, one-way ANOVA). In the PBS-treated group, 10 weeks post-treatment, NAA concentrations were significantly reduced (*P* = 0.027, ANOVA on ranks). In the HIgM12-treated group, we found a significant increase in NAA concentrations at both 5- and 10-week time points (*P* < 0.001, one-way ANOVA) (Figure 1B).

Analysis of pathology (cerebellum, cortex, brainstem, hippocampus, striatum, corpus callosum, and meninges) did not show any differences across groups (Additional file 1: Figure S1). Brainstem pathology was equivalent across groups (*P* = 0.51, ANOVA on ranks) (Figure 1C). Plotting individual NAA concentrations against corresponding brainstem pathological scores (Figure 1D) yielded no significant correlation (*P* = 0.59). We then analyzed the number of animals in each group with improved NAA concentrations 10 weeks after treatment. As a selection criterion, we used a minimum of ≥2 times the baseline standard error of the mean (SEM). NAA concentrations improved in 1 of the 10 mice in the control IgM-treated group, 1 of the 11 mice in the PBS-treated group, and 11 of the 13 mice in the HIgM12-treated group (Figure 1E). Fisher’s exact test showed this was highly significant (HIgM12 *vs* control IgM, *P* < 0.001; HIgM12 *vs* PBS, *P* < 0.001; and control IgM *vs* PBS, *P* = 0.39): 84% of mice treated with HIgM12 showed a positive response (Additional file 2: Figure S2).

**Figure 2:**
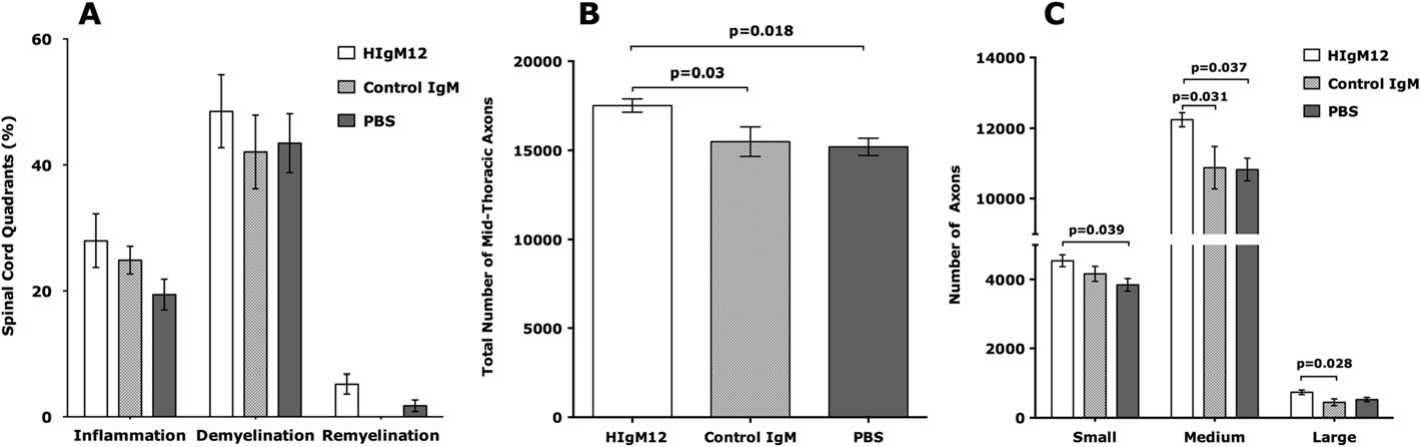
HIgM12 does not promote spinal cord remyelination but preserves spinal cord axons. **(A)** The same mice used to collect MR spectra longitudinally were sacrificed at 10 weeks post-treatment. Spinal cords were removed and processed for morphology analysis. Mice from all three treatment groups have similar levels of spinal cord inflammation, demyelination, and remyelination pathology. **(B)** When the total number of mid-thoracic level axons was compared across treatment groups, HIgM12-treated mice with improved NAA concentrations also contained more axons than the control IgM- and PBS-treated groups (*P* = 0.03 and *P* = 0.018 respectively, one-way ANOVA). **(C)** When axons of different calibers were analyzed, HIgM12-treated mice had more small-caliber (1 to 4 μm^2^, *P* = 0.039, one-way ANOVA) and medium-caliber (4 to 10 μm^2^, *P* = 0.037) axons than the PBS-treated mice. HIgM12-treated mice had more medium-caliber (4 to 10 μm^2^, *P* = 0.031) and large-caliber (>10 μm^2^, *P* = 0.028) axons than the control IgM-treated mice. Pathology analysis was performed blinded to the experimental groups.

We tested whether improved NAA concentrations in HIgM12-treated mice correlated with spinal cord pathology at the 10-week time point. Ten plastic-embedded cross sections from each mouse were scored for inflammation, demyelination, and remyelination. Pathological scores were similar across all three groups of mice (Figure 2A). We then counted axons from mid-thoracic (T6) spinal cord sections. Six areas encompassing approximately 400,000 μm^2^ of white matter were sampled from each mouse, and the total numbers of mid-thoracic axons were compared across treatment groups. HIgM12-treated mice with improved NAA concentrations contained more axons than the control IgM (17,524 ± 376 *vs* 15,488 ± 832) and PBS (17,524 ± 376 *vs* 15,198 ± 485) treated groups (Figure 2B). Detailed analysis of axons distribution revealed that HIgM12-treated mice had greater preservation of axons of all sizes including small-caliber (1 to 4 μm^2^, *P* = 0.039, one-way ANOVA), medium-caliber (4 to 10 μm^2^, *P* = 0.037), and large-caliber axons (>10 μm^2^, *P* = 0.028) (Figure 2C).

## Discussion

In this study, we demonstrate that a neuron-targeting human antibody is therapeutic in a progressive model of inflammatory demyelinating disease. It is generally very difficult to alter progression of neuropathology and neurologic deficits in the TMEV model. In the past, we documented that some human IgMs reactive to the surface of oligodendrocytes remyelinate spinal cord lesions in both the TMEV model of MS and in the lysolecithin-induced demyelination model [19,20]. Using retrograde tracing of demyelinated spinal cord axons, neuron cell bodies in the brainstem labeled with fluorescent markers indicating death or dysfunction were reduced [21]. This led to the concept to use brainstem NAA concentration as a sensitive marker to detect axon dysfunction well before it is evident histologically [12].

Here, we examined whether treatment with neuron-binding human antibody, HIgM12, alters brainstem NAA concentrations in SJL mice at the stage of maximal demyelination prior to significant axonal loss. A single administration of HIgM12 given at 90 dpi improved brainstem NAA concentrations significantly at both 5 and 10 weeks post-treatment. It is important to note that at the 10-week time point brainstem pathological scores were equivalent across groups, suggesting that brainstem pathology had no influence on NAA concentrations. We clearly find that a single administration of HIgM12 is sufficient for improved brainstem NAA concentrations in the TMEV model, but a single dose may not be the optimum treatment. Treatment with multiple doses of HIgM12 in animal models is however hampered due to strong anti-human antibody responses with subsequent administrations that likely inactivate HIgM12 in circulation. The human IgM used in this study is available as a GLP grade product suitable for translation to human trials. Multiple doses of HIgM12 to treat human neurodegeneration may significantly enhance efficacy in patients greater than in disease models’.

We previously showed that a consistent 1-μm cross section from the spinal cord can be used for axon frequency analysis [15]. Using mid-thoracic sections, we found that HIgM12-treated group in this study had more axons of all calibers. Upon further analysis, we determined that medium-caliber axons, which represent the largest population of axons, were primarily preserved. We also found significant differences in the small- and large-axon bins between the HIgM12 and PBS groups and between HIgM12 and control IgM, respectively. One possible explanation for greater numbers of small- and medium-caliber axons is large-caliber axons reduce their diameter due to atrophy. This finding supports our previous observation of shrinking of large-caliber axons in two independent studies in different mouse strains [22,23].

Our data also suggests that demyelination may be a necessary, but insufficient condition for neurologic deficits associated with demyelinating diseases. The primary goal of neuroprotection is to sustain the functional integrity of neurons and limit dysfunction. MR spectroscopy and axon-count analyses provide strong evidence that HIgM12 affected neuronal viability through the preservation of axons, despite equivalent levels of demyelination across treatment groups. Likewise, perforin-deficient mice with a similar degree of demyelination also had significantly more mid-thoracic axons compared to wild-type littermate controls [16]. Our findings are consistent with axonal damage occurring independently of chronic demyelination. This concept was suggested by several studies of gray matter lesions and meningeal infiltrates in both human brain and genetically manipulated mice [24-28].

In conclusion, our results provide the first evidence for antibody-mediated axon protection in a mouse model of progressive MS. The precise mode of action of the antibody is currently under investigation. We hypothesize that treatment with HIgM12 directly protects spinal cord axons and indirectly protects neurons in the brainstem, which may underlie brainstem NAA concentrations.

## Additional files

Additional file 1: Figure S1. Brain pathology was similar across treatment groups. Individual brain pathology scores in all three treatment groups were collected without knowledge of the treatment groups. Brain pathology was quantified using five-point grading system. Overall pattern of individual pathological scores from different brain regions showed no major differences among the three treatment groups. Statistical comparison revealed no differences (*P* = 0.144, one-way Kruskal-Wallis ANOVA). Additional file 2: Figure S2. Majority of HIgM12-treated mice show improved NAA concentrations in the brainstem. Individual NAA concentrations were calculated at three different time points: baseline, 5 weeks, and 10 weeks post-treatment. Eleven of the 13 mice (84.6%) in the HIgM12-treated group showed an upward trend for NAA concentrations at 5- and 10-week time points, whereas only 1 mouse per group showed improved NAA concentrations in the control groups (*N* = 10, control IgM; *N* = 11, PBS).

## Abbreviations

CNS: Central nervous system
HIgM12: Human immunoglobulin M 12
MRS: Magnetic resonance spectroscopy
MS: Multiple sclerosis
NAA: *N*-acetyl-aspartate
PPMS: Primary progressive multiple sclerosis
RRMS: Relapsing remitting multiple sclerosis
SC: Spinal cord
SJL: Swiss Jim Lambert
SPMS: Secondary progressive multiple sclerosis
TMEV: Theiler’s murine encephalomyelitis virus
