# Supplementary figures and images for: A single dose of a neuron-binding human monoclonal antibody improves brainstem NAA concentrations, a biomarker for density of spinal cord axons, in a model of progressive multiple sclerosis
Source: J Neuroinflammation. 2015 Apr 29;12:83. doi: 10.1186/s12974-015-0303-y (PMC4418041; doi:10.1186/s12974-015-0303-y)

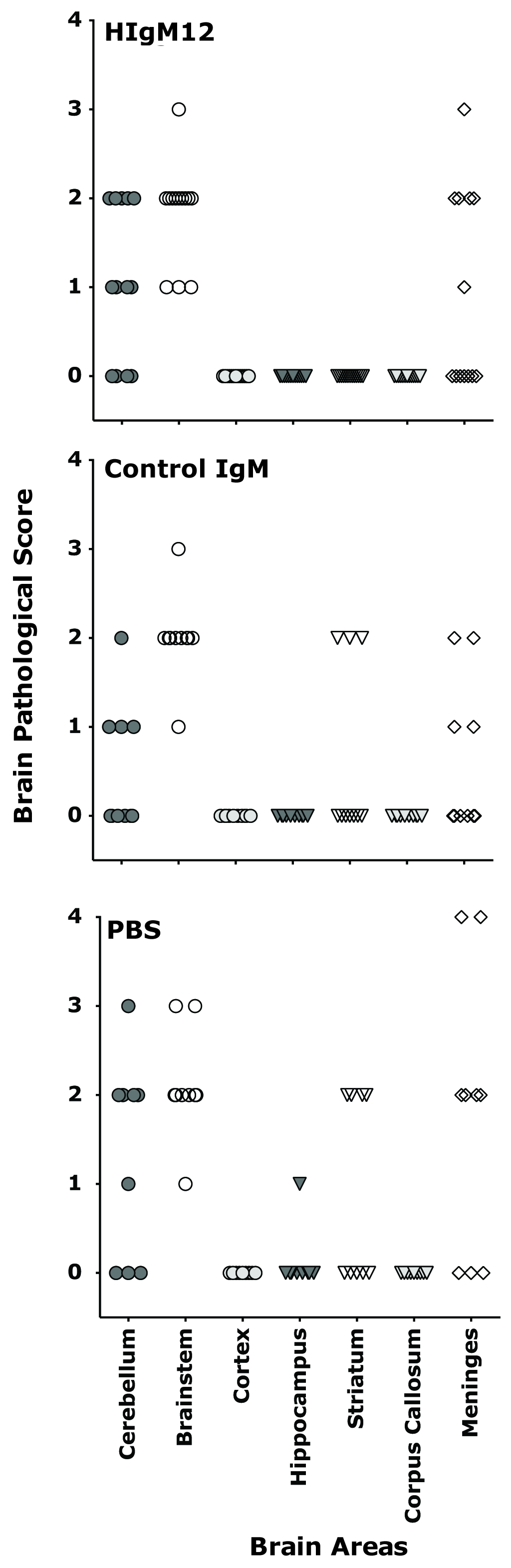

Supplement: Additional file 1: Figure S1. — Brain pathology was similar across treatment groups. Individual brain pathology scores in all three treatment groups were collected without knowledge of the treatment groups. Brain pathology was quantified using five-point grading system. Overall pattern of individual pathological scores from different brain regions showed no major differences among the three treatment groups. Statistical comparison revealed no differences (P = 0.144, one-way Kruskal-Wallis ANOVA). [file 12974_2015_303_MOESM1_ESM.tif]

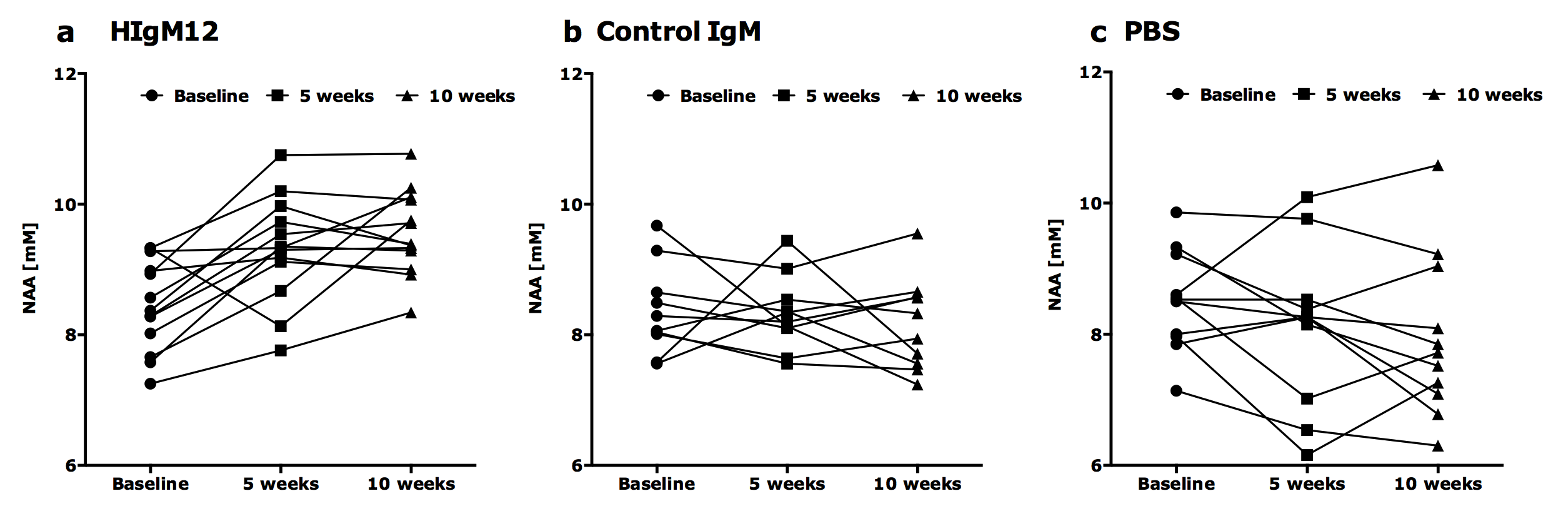

Supplement: Additional file 2: Figure S2. — Majority of HIgM12-treated mice show improved NAA concentrations in the brainstem. Individual NAA concentrations were calculated at three different time points: baseline, 5 weeks, and 10 weeks post-treatment. Eleven of the 13 mice (84.6%) in the HIgM12-treated group showed an upward trend for NAA concentrations at 5- and 10-week time points, whereas only 1 mouse per group showed improved NAA concentrations in the control groups (N = 10, control IgM; N = 11, PBS). [file 12974_2015_303_MOESM2_ESM.tiff]
